# Monitoring Dengue Virus in *Aedes aegypti* to Improve Dengue Surveillance and Control in Puerto Rico

**DOI:** 10.3390/v17121539

**Published:** 2025-11-25

**Authors:** Luisa M. Otero, Joanelis Medina, Jose Ruiz-Valcarcel, Reinaldo Rivera, Yashira Maldonado, Jomil Torres, Zachary J. Madewell, Laura Adams, Gabriela Paz-Bailey, Roberto Barrera

**Affiliations:** 1 Entomology and Ecology Team, Dengue Branch, Division of Vector-Borne Diseases, Centers for Disease Control and Prevention, San Juan 00920, Puerto Rico; nrs4@cdc.gov (L.M.O.); pmn3@cdc.gov (J.R.-V.); rov3@cdc.gov (R.R.); svz3@cdc.gov (Y.M.); ock0@cdc.gov (Z.J.M.); ipb2@cdc.gov (L.A.); gmb5@cdc.gov (G.P.-B.); 2 Puerto Rico Vector Control Unit, San Juan 00927, Puerto Rico; jomedina@prvectorcontrol.org; 3 Department of Health of Puerto Rico, San Juan 00936, Puerto Rico; jomil.torres@salud.pr.gov

**Keywords:** entomo-virological surveillance, *Aedes aegypti*, dengue virus, dengue, vector control

## Abstract

*Aedes aegypti* is the primary urban vector for several important arboviruses, including dengue, chikungunya, yellow fever, and Zika viruses. Traditional dengue virus (DENV) surveillance relies on passive reporting of human cases, which often underestimates transmission due to asymptomatic or unreported infections. This study evaluated the utility of monitoring DENV in *Ae. aegypti* mosquitoes to improve detection of local dengue transmission and inform vector control strategies during the 2024 dengue epidemic in Puerto Rico. Mosquito surveillance was conducted in 15 neighborhoods within the San Juan metropolitan area where confirmed dengue cases had been recently reported. Adult female *Ae. aegypti* were collected weekly using Autocidal Gravid Ovitraps (AGO traps) placed within a 200 m radius of index cases. Pools of 1–20 mosquitoes were tested for DENV RNA and serotype using RT-PCR. Surveillance continued for up to 91 days in study areas, depending on virus detection. A total of 29,354 female *Ae. aegypti* were collected, of which 29,211 females were pooled (1–20 specimens per pool) into 3878 pools and analyzed. DENV was detected in 49 pools across 11 neighborhoods, with serotypes DENV-1, DENV-2, and DENV-3 identified. Multiple serotypes were sometimes detected in mosquitoes from the same neighborhood. Minimum infection rates and vector indices were higher during the epidemic than in previous inter-epidemic periods, and mosquito densities exceeded thresholds considered protective against outbreaks. Entomo-virological surveillance detected a greater variety and evenness of serotypes than passive human surveillance. These findings suggest that entomo-virological surveillance can complement passive case surveillance, providing a more comprehensive detection of DENV circulation. Integrating mosquito-based and human surveillance can improve outbreak detection, guide vector control, and aid in reducing dengue burden in affected communities.

## 1. Introduction

*Aedes aegypti* is the primary urban vector for several significant arboviruses, including dengue, chikungunya, yellow fever, and Zika viruses [1]. Current control strategies rely on mosquito management guided by passive disease surveillance, which relies on reporting from healthcare providers and laboratories. While disease surveillance is essential for planning responses and alerting the public, it may not fully capture transmission, especially for arboviruses like dengue and Zika, where many infections are asymptomatic or unreported [2,3,4,5]. Asymptomatic individuals can still infect mosquitoes, making undetected cases a challenge for effective vector control [6]. Studies have also indicated that high dengue incidence can occur in endemic areas that are not identified through traditional surveillance [7,8]. Therefore, relying solely on passive surveillance for reactive vector control is insufficient to stop transmission of viruses like dengue and Zika [9]. Previous studies suggest that entomo-virological surveillance can provide valuable insights into the dynamics of dengue and Zika transmission [10,11]. However, reactive vector control is resource-intensive, and responding to all virus detections and cases once transmission is generalized has been shown to be unfeasible.

Entomo-virological surveillance can help determine how long arboviruses circulate in a neighborhood after being introduced by an infected person. Detecting viruses in locally collected *Ae. aegypti* is particularly relevant because this mosquito has a limited dispersal range [12], and within this area, females seek blood meals from nearby hosts. Thus, the presence of arboviruses in *Ae. aegypti* indicates the presence of somebody with an infection nearby, unless the virus comes about from vertical transmission. Indirect pathogen detection in vectors can strengthen surveillance and control efforts, especially since collecting blood samples from asymptomatic or inapparently infected people is resource-intensive. Another limitation of passive surveillance is that dengue cases are reported by the patient’s residence, which does not always reflect the actual site of infection. Nevertheless, some studies showed that a large fraction of infections occur at home or nearby. For example, a contact tracing study in Cairns, Australia, found that over 42.8% of dengue cases were exclusively linked to the patient’s home [13]. Another study conducted in Thailand revealed that 60% of dengue cases located less than 200 m apart came from the same DENV transmission chain [14].

Neighborhood-scale entomo-virological studies in Puerto Rico during the chikungunya and Zika epidemics detected these viruses in local *Ae. aegypti* populations for five to eight consecutive weeks. After this period, virus detections were sparse, suggesting that later cases likely resulted from new, separate virus introductions [15]. Adult *Ae. aegypti* mosquitoes collected at homes of dengue patients were positive for up to three weeks since onset of symptoms in Merida, Mexico [16]. Determining how long *Ae. aegypti* can locally transmit arboviruses in urban communities is important for guiding public health actions, such as enhanced surveillance and vector control [17,18]. This information is especially valuable when a new or reemerging arbovirus is detected, enabling timely mosquito control to prevent further spread. Monitoring arboviruses in *Ae. aegypti* also provides data on mosquito abundance and infection rates during outbreaks and helps assess the effectiveness of control measures [15,19,20,21,22,23].

Puerto Rico experienced a dengue epidemic of serotypes DENV-1, DENV-2, and DENV-3 in 2024, with 6291 cases [24]. In this investigation, we monitored the abundance of adult female *Ae. aegypti* and their minimum infection rates for several weeks in each of several neighborhoods where dengue cases had been confirmed. The main objectives were 1—to determine how long dengue viruses circulated locally to make recommendations on vector control, 2—to characterize the DENV serotypes present, and 3—to compare the occurrence of DENV in mosquitoes with the number of passively reported dengue cases among residents of the same neighborhoods.

## 2. Materials and Methods

To investigate the presence of dengue viruses in mosquitoes following a confirmed dengue case reported to the Puerto Rico Department of Health in a resident of the study neighborhoods, we installed mosquito traps within a 200 m radius of the case. These traps were used to capture female *Ae. aegypti* mosquitoes, document their relative abundance (females per trap per week), collect pools of 1–20 female mosquito specimens per trap for virus identification, and track subsequent dengue cases reported in the neighborhood, which could have resulted from local transmission or introductions.

### 2.1. Study Area

Fieldwork was conducted successively from December 2023 to December 2024 in the 200-m study areas around confirmed cases in 15 neighborhoods within the San Juan metropolitan area (Carolina, Cataño, Guaynabo, and San Juan municipalities). The neighborhoods studied were residential areas with 216–519 houses each (Supplemental Table S1).

### 2.2. Dengue Case Data

Dengue is a notifiable disease in Puerto Rico. Confirmed dengue cases were defined as a positive result for detection of DENV nucleic acid by reverse transcriptase-polymerase chain reaction (PCR) [25]. The Puerto Rico Health Department provided information on symptoms onset of initial and subsequent confirmed dengue cases in the study areas, virus serotype, and approximate location of residence. The precise location of dengue cases was not available to field personnel. Although we refer to initial confirmed dengue cases, we could not determine whether a case represented a recent introduction of DENV into the neighborhood or if the confirmed case was merely the “tip of the iceberg,” resulting from ongoing, silent transmission. Surveillance was conducted in neighborhoods as soon as the location of a confirmed dengue case and its approximate location were available (Table S1).

### 2.3. Ethics Statement

In this study, we use data on *Ae. aegypti* mosquitoes and the detection of dengue viruses within these vectors. Our study did not involve human subjects, including the collection of data or biological samples from individuals. Therefore, this study did not need Institutional Review Board (IRB) review.

### 2.4. Entomological Surveillance

Female *Ae. aegypti* adults were monitored every week using 30 Autocidal Gravid Ovitraps (AGO traps) [26] per neighborhood that were placed outdoors in houses that agreed to participate in the study. The traps were evenly distributed across the circular 200 m radius area around the confirmed dengue case, maximizing the inclusion of buildings and avoiding large uninhabited spaces and highways. Mosquito collections were conducted for a minimum period of three weeks if no positive pools were detected, and mosquito surveillance stopped once there were two consecutive weeks without positive pools, although some of these periods were extended due to delays in the analyses of pools (Table S1).

### 2.5. Detecting Dengue Viruses in Ae. aegypti

Female *Ae. aegypti* mosquitoes collected from individual AGO traps were pooled weekly into groups of 1–20 mosquitoes per vial and preserved at −20 °C until processed using the CDC’s trioplex RT-PCR assay to detect viral RNA of dengue, chikungunya, and Zika viruses [27,28]. Previous studies have shown that viral RNA of DENV, CHIKV, and ZIKV can be detected in dead mosquitoes exposed to room temperature or under field conditions for over a week [15]. To prevent cross-contamination, dissecting needles were sterilized between transfers from individual traps to vials. A super pool approach was used to enhance testing efficiency [29]. We combined 50 µL aliquots from each of the ten individual pools into a single 500 µL super pool sample. Positive super pools prompted additional testing by RT-PCR of each of the ten individual pools to identify which of the individual pools had the virus and to determine the dengue serotype (DENV-1, DENV-2, DENV-3, or DENV-4) [28].

### 2.6. Statistical Methods

Minimal infection rates (MIR; the proportion of positive pools out of the total number of processed mosquitoes, multiplied by one thousand) were calculated using bias-corrected maximum likelihood methods with the “PooledInfRate” 1.6 package in R (https://github.com/CDCgov/PooledInfRate (accessed on 9 September 2025). The vector index (VI) represents the expected number of infected mosquitoes per one thousand mosquitoes and is calculated as the product of the infection rate and the average density of mosquitoes, multiplied by one thousand.

To explore the relationship between the density of female *Ae. aegypti* and the probability of virus positivity, we conducted a Generalized Linear Mixed Model (GLMM) with a binomial distribution and a logit link function of DENV positivity (positive = 1, negative = 0) against mosquito density per trap per week and included neighborhood as a random variable. We used the library lme4 in R. We used a two-sided Mann–Whitney U test (α = 0.05) to compare the median abundance of *Ae. aegypti* between samples with positive and negative pools.

The number of DENV detections per month was used to compare the sensitivities of entomo-virological (positive pools of *Ae. aegypti*) and human dengue (initial and subsequent reported dengue cases) surveillance approaches during the study. We calculated the Shannon diversity H = −∑(pi × ln(pi)) and Pielou’s evenness (J = H/ln S) indices [30], where p_i_ is the proportion of each DENV serotype detected (DENV-1, DENV-2, DENV-3) and S is the number of serotypes (3). The diversity index reaches higher values with a greater number of species (serotypes) and with a more even distribution of each serotype in the sample (months). Evenness measures how evenly distributed the abundance of each serotype is in the samples and varies from 0 to 1. We interpret that a surveillance system with higher diversity and evenness indices demonstrates better sensitivity to the presence and abundance of DENV serotypes in the samples. We used the vegan package in R to calculate the indices.

## 3. Results

**Figure 1:**
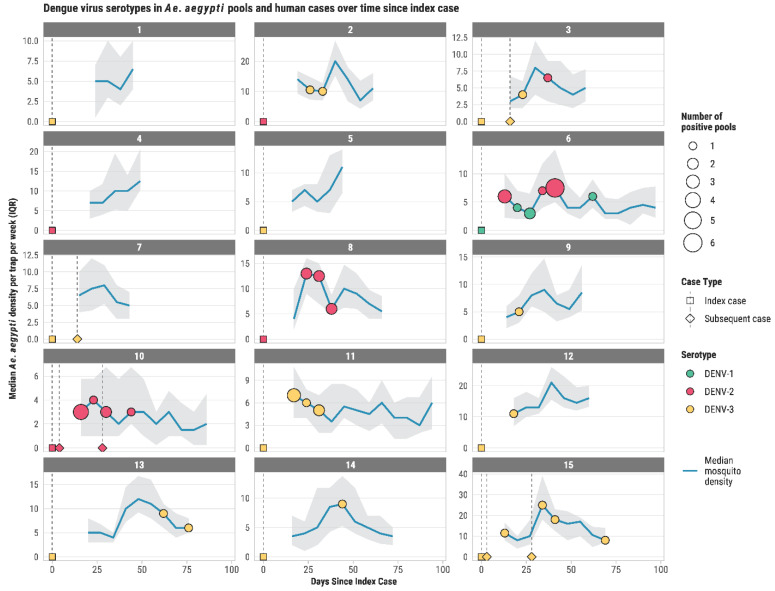
Dengue virus serotypes in *Ae. aegypti* pools and human cases in 200-m radius areas around confirmed dengue cases in each of 15 neighborhoods monitored from December 2023 to December 2024 in San Juan, Puerto Rico. The median density of female *Ae. aegypti* density per trap per week is shown as a blue line, with shaded bands indicating the interquartile range (IQR). Colored circles represent mosquito pools positive for dengue virus, sized by the number of positive pools and colored by serotype. Human cases are shown on day 0 (index case) or on subsequent days after the index case, represented by squares (index cases) and triangles (subsequent cases), also colored by serotype. Dashed gray lines indicate the timing of reported cases. This figure highlights concordance and mismatch between dengue virus serotypes detected in mosquitoes and those in human cases across the study areas.

Mosquito surveillance was initiated in the 15 study neighborhoods 7 to 22 days (mean ± SE: 10 ± 1 days) after receiving notification of a confirmed dengue case and was conducted for 24 to 91 days, depending on the detection of positive pools (Table S1). During the study, we captured 29,354 female *Ae. aegypti* mosquitoes in AGO traps (N = 3514 trap-weeks), with average (±SE) numbers per neighborhood ranging from 3.6 ± 0.2 to 16.0 ± 0.6 mosquitoes per trap per week (Table 1). Of the total captures, 29,211 specimens were pooled into 3878 individual pools that were analyzed by RT-PCR to detect the presence of DENV RNA and to identify the serotypes. We found 49 (49/3878; 1.3%) dengue-positive pools in 11 out of 15 (73%) neighborhoods (Figure 1). Among the serotypes identified, 6 were DENV-1, 25 DENV-2, and 18 DENV-3). Positive pools were detected as early as the first week of mosquito surveillance in two neighborhoods, and detections occurred in some neighborhoods for 2 to 5 consecutive weeks (Figure 1). The number of positive pools per week within the same neighborhood varied from 1 to 6, as observed in neighborhood # 6 (Figure 1).

**Figure 2:**
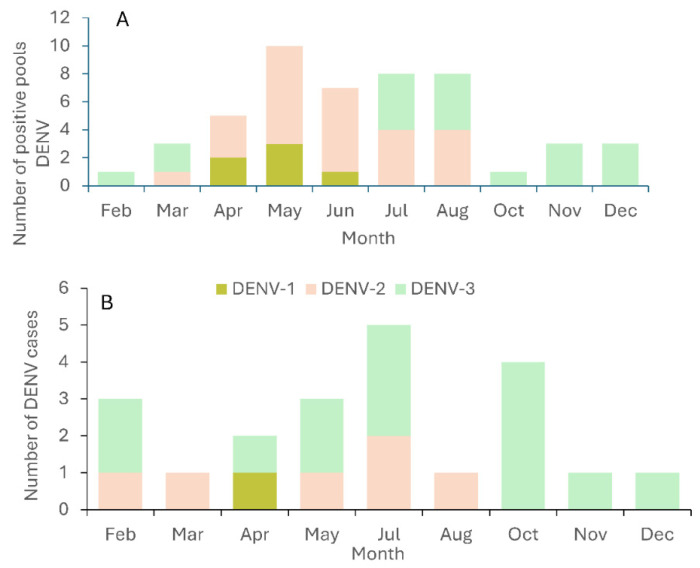
Number of dengue-positive female *Ae. aegypti* pools (**A**) and number of confirmed human dengue cases reported in the passive surveillance system (**B**) by serotype per month in San Juan, Puerto Rico.

Dengue virus serotypes identified in both human cases and *Ae. aegypti* mosquitoes were DENV-1, DENV-2, and DENV-3 (Figure 2). Only DENV-3 was observed in both human cases and mosquitoes during the last three months of the study (October–December 2024). The DENV serotypes of the 15 initial (index) dengue cases were DENV-1 (one case), DENV-2 (four cases), and DENV-3 (ten cases). The passive dengue surveillance system reported six subsequent dengue cases in four neighborhoods, all of which were of the same serotype as the initial dengue cases in those neighborhoods (2 DENV-2 and 4 DENV-3; Table 2). The DENV serotype of the initial human dengue case was also detected in mosquitoes collected in the same areas. However, on several occasions, mosquitoes from those neighborhoods also carried other DENV serotypes (Table 2). This indicates that more than one dengue serotype was introduced and possibly circulated in the same neighborhoods during the observation period. Furthermore, the presence of dengue viruses in mosquitoes in the absence of subsequent human cases in most neighborhoods provides evidence of broad silent virus circulation.

Minimum infection rates (MIR) in mosquito pools varied from 0.30 to 7.52 per thousand, and vector indices (VIs) ranged from 3.75 to 42.86 per thousand (Table 1). The median number of female *Ae. aegypti* per trap per week was 9 (mean ± SE = 12.7 ± 1.5) during weeks when positive pools were detected, and 6.5 (8.3 ± 0.1) during weeks when no positive pools were detected; this difference was significant (Mann–Whitney U = 110,278.5, *p* < 0.001).

Results of the GLMM of DENV pool positivity in relation to mosquito density per trap per week showed significant effects (b = 0.082; 95% CI = 0.036–0.126; z = 3.59; *p* < 0.001), indicating a positive association between the number of captured mosquitoes and the probability that pools of these mosquitoes tested positive for DENV. The odds ratio (1.086) shows that, for each additional mosquito captured in AGO traps, the odds of the pool being positive for DENV increased by about 8.6% (3.7–13.5%). There was a small decrease in deviance from 525.8 to 513.6, indicating that mosquito density explained only a small portion of the variance in pool positivity.

The Shannon index of serotype diversity was higher in pooled samples of *Ae. aegypti* (0.98) than in reported human cases (0.77). Similarly, serotype evenness was greater in mosquitoes (0.88) than in human cases (0.70). These results indicate that entomo-virological surveillance in mosquitoes provided more comprehensive information about circulating DENV serotypes in the neighborhoods investigated than data obtained from the passive dengue surveillance system (Figure 2).

## 4. Discussion

Arboviruses are presumed to circulate for only a limited period at the local or neighborhood level due to the depletion of susceptible hosts. However, there is little information in the literature regarding duration of local arbovirus transmission despite its importance for tracking disease cases, implementing effective vector control, and informing modeling efforts. The primary questions of this investigation were whether monitoring dengue viruses in local *Ae. aegypti* females could provide insights into local transmission dynamics and the duration of transmission following the report of an initial confirmed case. This approach also allowed us to compare the effectiveness of detecting dengue viruses in mosquitoes with dengue case reports collected through a passive surveillance system in Puerto Rico during the 2024 dengue epidemic.

Our results showed the presence of dengue viruses in *Ae. aegypti* mosquitoes in most of the neighborhoods investigated, which contrasted sharply with the limited number of dengue cases reported in the passive surveillance system after reports of an initial case during the period under investigation. These findings indicate that there was significant silent or inapparent transmission of dengue viruses—a phenomenon broadly documented in previous studies [31,32]. Silent or inapparent circulation of dengue viruses is expected as only one fourth of cases are estimated to be symptomatic, and most infections are mild; therefore, even if a person is symptomatic, they may not seek care. The percentage of symptomatic infections varies by previous immunity and serotype, with studies suggesting a smaller percentage of primary DENV-2 infections being symptomatic compared to DENV-3 [33]. This would align with our findings, as DENV-2 was the most frequently detected virus in mosquito pools, while DENV-3 human cases were more frequently reported.

The results also revealed the presence of DENV in *Ae. aegypti* over consecutive weeks in several neighborhoods, indicating uninterrupted transmission for at least five weeks. Because mosquito surveillance began with a 2–3-week lag, the actual period of continuous transmission may have been longer than five weeks. However, if the confirmed dengue case was the “tip of the iceberg”—meaning that dengue transmission was already ongoing for a while—then observing positive pools of DENV in mosquitoes for consecutive weeks would be less likely. Similar periods of consecutive weekly virus detections in *Ae. aegypti* have been observed for chikungunya and Zika viruses in a previous study in southern Puerto Rico [15]. These findings underscore the challenges of reactive mosquito control strategies and suggest that enhanced surveillance, control measures, and community outreach should be sustained for at least two months and implemented across the entire neighborhood. In the context of silent transmission, this strategy becomes particularly important when a new or reemerging arbovirus is introduced into a neighborhood, ensuring that active case finding and vector control are maintained and applied community-wide for several weeks [34].

The simultaneous circulation of DENV-1, DENV-2, and DENV-3 during the 2024 epidemic complicates our understanding of transmission dynamics. In most neighborhoods with positive mosquito pools (8 out of 11), the DENV serotypes detected in mosquitoes matched those identified in the putative index case. However, in three neighborhoods, different serotypes were found in mosquitoes, indicating the introduction of additional serotypes. In one neighborhood, two different serotypes were circulating simultaneously. These results demonstrate frequent introductions of dengue viruses into local communities, which may intensify local transmission and increase the risk of severe dengue. Since most dengue infections are asymptomatic, passive dengue case surveillance, which relies on patients seeking care and clinicians ordering diagnostic tests, may not provide an accurate picture of circulating serotypes [8]. Similar findings were reported in a study of dengue serotype occurrence in patients and mosquitoes, where not all mosquito pools had the same dengue serotype as the patient, and one out of three circulating serotypes was found only in mosquitoes [23].

The likelihood of finding positive pools of *Ae. aegypti* was positively correlated with mosquito density, although the percentage of variance explained by this variable was small, suggesting the importance of other factors not included in the study. Notably, the densities of *Ae. aegypti* in AGO traps in all study neighborhoods were above the thresholds considered protective against rampant local outbreaks of arboviruses in Puerto Rico: less than four individuals/trap/week for dengue and less than three individuals/trap/week for chikungunya and Zika [15,20,35]. One exception was a positive pool found in one neighborhood during a week, with 3.7 individuals/trap/week. Using these density threshold values as a reference for protection against local outbreaks caused by these viruses, it is evident that the density of *Ae. aegypti* in the studied neighborhoods was above those levels. These observations indicate that the studied neighborhoods were receptive to virus transmission with respect to both vector density and the rate of contact with susceptible hosts.

Other metrics included in the study were the minimum infection rate (MIR) and vector index (VI), which measure the prevalence of dengue viruses in *Ae. aegypti* females and the expected number of infected mosquitoes per thousand mosquitoes, respectively. However, comparisons of these metrics across arbovirus epidemics and their utility as indicators of transmission risk are difficult to interpret unless they have been estimated using the same methodology (e.g., mosquito collection technique, sample size, analytical methods). In Puerto Rico, using the same methodology, dengue infection rates of female *Ae. aegypti* in 2024 were higher (0.30–7.52) than in a previous study (0.13–0.33) conducted during an inter-epidemic period (2020–2023) [20]. Interestingly, dengue infection rates of *Ae. aegypti* during this dengue epidemic were lower than those observed during the epidemics of chikungunya (6.9–12.1) and Zika (4.7–13.7) in southern Puerto Rico [15]. Similarly, VI values for the 2024 dengue epidemic (3.8–42.9) were higher than during the previous inter-epidemic period (1.0–2.9), but lower than during the chikungunya (42–75) and Zika (29–85) epidemics. There appears to be a correspondence between rates of arbovirus infections in *Ae. aegypti* and the severity of epidemics. There were 6291, 28,327, and 39,000 cases of dengue (2024), chikungunya (mostly in 2014), and Zika (mostly in 2016) epidemics in Puerto Rico, respectively [24,36,37]. Similar associations between dengue infections in *Ae. aegypti* and the severity of dengue epidemics have been reported previously [38]. Thus, these metrics of arbovirus prevalence in *Ae. aegypti* may be useful indicators for evaluating the intensity of virus transmission and epidemic potential.

One limitation of this study was the use of reported, confirmed dengue cases as triggers for entomological surveillance. Ideally, we would use the first DENV infection introduced into the neighborhood as the index case. However, given the high proportion of asymptomatic or non-specific infections, this approach would require extensive testing of residents. While such comprehensive testing is possible, it would be logistically challenging. Surveillance of dengue viruses in local *Ae. aegypti* is a form of active surveillance that increases the likelihood of finding the virus and complements passive case surveillance in the absence of active infection surveillance in people.

## 5. Conclusions

This study demonstrates that monitoring dengue viruses in local *Ae. aegypti* females provides valuable insights into the dynamics and duration of dengue transmission at the neighborhood level. Our findings revealed that dengue viruses were present in *Ae. aegypti* mosquitoes in most neighborhoods studied, even where few or no dengue cases were reported through passive surveillance. This highlights substantial silent or inapparent transmission, which complicates efforts to anticipate severe dengue cases and underscores the limitations of relying solely on case-based surveillance and reactive vector control. The reports of dengue cases through a passive surveillance system may have led to an incomplete picture of the actual disease burden in the study areas. Finally, given that dengue viruses circulated for at least six weeks in these neighborhoods, *Ae. aegypti* control should be sustained for that long to prevent local infections and virus export to other urban areas.

## Figures and Tables

**Table 1:** Serotypes of initial dengue cases, pools of mosquitoes, and subsequent dengue cases in 15 neighborhoods of the San Juan Metropolitan Area, Puerto Rico (December 2023–December 2024).

Neighborhood	Initial Case DENV Serotype	Subsequent Dengue Cases and Their Serotypes	Positive Mosquito Pools and Serotypes		
			DENV-1	DENV-2	DENV-3
1	DENV-3	0	0	0	0
2	DENV-2	0	0	0	2
3	DENV-3	1 DENV-3	0	1	1
4	DENV-2	0	0	0	0
5	DENV-3	0	0	0	0
6	DENV-1	0	6	10	0
7	DENV-3	1 DENV-3	0	0	0
8	DENV-2	0	0	6	0
9	DENV-3	0	0	0	1
10	DENV-2	2 DENV-2	0	8	0
11	DENV-3	0	0	0	6
12	DENV-3	0	0	0	1
13	DENV-3	0	0	0	20
14	DENV-3	0	0	0	1
15	DENV-3	2 DENV-3	0	0	4
Total	15	6	6	25	18

**Table 2:** Female *Aedes aegypti* density (individuals/trap/week), infection rates with DENV, and Vector Index by neighborhood from December 2023 to December 2024 in San Juan City, Puerto Rico.

Neighborhood	Total Pools Analyzed	Positive Pools	Analyzed Mosquitoes	Average Mosquito Density (±S.E.)	Pool Infection Rate (95% C.I.)	Vector Index (95% C.I.)
1	170	0	634	6.1 ± 0.5	0	0
2	361	2	2739	13.7 ± 0.7	0.73 (0.13–2.39)	10.00
3	332	2	1246	6.1 ± 0.4	1.61 (0.29–5.24)	9.82
4	167	0	1720	11.1 ± 0.8	0	0
5	154	0	1220	7.8 ± 0.5	0	0
6	366	16	2191	5.7 ± 0.2	7.52 (4.47–11.92)	42.86
7	151	0	1105	7.4 ± 0.4	0	0
8	248	6	2291	9.6 ± 0.5	2.67 (1.09–5.54)	25.63
9	211	1	1637	7.8 ± 0.4	0.61 (0.04–2.95)	4.76
10	278	8	1187	3.6 ± 0.2	6.89 (3.23–13.05)	24.80
11	338	6	2060	5.7 ± 0.2	2.94 (1.2–6.08)	16.78
12	255	1	3303	16.0 ± 0.6	0.30 (0.02–1.47)	4.80
13	265	2	2184	8.2 ± 0.3	0.92 (0.16–2.99)	7.54
14	264	1	1773	6.7 ± 0.3	0.56 (0.03–2.72)	3.75
15	318	4	3921	15.1 ± 0.7	1.03 (0.33–2.47)	15.55
Total	3878	49	29,211	8.4 ± 0.13	-	-

## Data Availability

Data are contained within the article and Supplemental Materials.

## Supplementary Materials

The following supporting information can be downloaded at: https://www.mdpi.com/article/10.3390/v17121539/s1, Table S1, Description of the neighborhoods where the study took place in the metropolitan area of San Juan, Puerto Rico, showing number pf houses per neighborhood, dates when the first confirmed dengue case was detected and located, days after the date when entomo-virological surveillance in *Ae. aegypti* began, and duration of mosquito and virus surveillance.
