# Supplementary material for: Monitoring Dengue Virus in Aedes aegypti to Improve Dengue Surveillance and Control in Puerto Rico
Source: Viruses. 2025 Nov 25;17(12):1539. doi: 10.3390/v17121539 (PMC12737552; doi:10.3390/v17121539)
Supplement: Supplementary file 1 [file viruses-17-01539-s001.zip › viruses-3934142-supplementary.pdf]

Table S1. Description of the neighborhoods where the study took place in the metropolitan area of San Juan, Puerto Rico, showing number pf houses per neighborhood, dates when the first confirmed dengue case was detected and located, days after the date when entomo-virological surveillance in *Ae. aegypti* began, and duration of mosquito and virus surveillance.

| Neighborhood | Municipality | Houses | Confirmed case date | Days after confirmed case | Duration of surveillance (days) |
|---|---|---|---|---|---|
| 1 | San Juan | 281 | 31 Dec 2023 | 22 | 24 |
| 2 | San Juan | 315 | 2 Feb 2024 | 14 | 48 |
| 3 | San Juan | 227 | 12 Feb 2024 | 10 | 49 |
| 4 | San Juan | 334 | 27 Mar 2024 | 13 | 35 |
| 5 | Carolina | 233 | 8 Apr 2024 | 7 | 35 |
| 6 | San Juan | 219 | 11 Apr 2024 | 7 | 91 |
| 7 | Carolina | 272 | 14 May 2024 | 10 | 35 |
| 8 | San Juan | 300 | 19 May 2024 | 9 | 54 |
| 9 | San Juan | 260 | 3 Jul 2024 | 9 | 49 |
| 10 | San Juan | 241 | 15 Jul 2024 | 7 | 77 |
| 11 | Carolina | 234 | 14 Jul 2024 | 8 | 87 |
| 12 | San Juan | 401 | 20 Jul 2024 | 10 | 49 |
| 13 | Cataño | 285 | 10 Oct 2024 | 11 | 63 |
| 14 | San Juan | 216 | 14 Oct 2024 | 8 | 63 |
| 15 | San Juan | 519 | 17 Oct 2024 | 7 | 63 |
